# Pneumopericardium, a Heart in a Trap

**DOI:** 10.3390/jcm13247636

**Published:** 2024-12-15

**Authors:** Małgorzata Rak, Oskar Fogiel, Marcos Chesi, Michał Starosolski, Tomasz Ilczak, Adam Śmiałowski, Anna Krakowiak

**Affiliations:** 1 Department of Emergency Medical Service, Faculty of Medicine, Silesian Academy in Katowice, 40-555 Katowice, Poland; malgorzatarak999@gmail.com; 2 Student Scientific Society, Department of Emergency Medicine, Medical University of Silesia, 40-055 Katowice, Poland; oskarfogiel0908@gmail.com; 3 Cooperativa de Trabalho dos Medicos do Hospital das Clinicas da UFMG, Belo Horizonte 30130-100, Brazil; marcoschesi@gmail.com; 4 Department of Emergency Medicine, Faculty of Medical Sciences in Katowice, Medical University of Silesia, 40-055 Katowice, Poland; 5 Department of Emergency Medicine, Faculty of Medical Sciences, University of Bielsko-Biala, 43-300 Bielsko-Biala, Poland; tilczak@ath.bielsko.pl; 6 Department of Endocrynology and Metabolic Diseases, Medical University of Lodz, 90-419 Lodz, Poland; adam.smialowski@umed.lodz.pl; 7 Clinic of Anaesthesiology and Intensive Care, Central Clinical Hospital, Medical University of Lodz, 92-213 Lodz, Poland; a.krakowiak@csk.umed.pl

**Keywords:** pneumopericardium, tension pneumothorax, diagnostic imaging, damage control, FAST, CT

## Abstract

**Background:** The text discusses the case of a patient who experienced pneumopericardium because of a traumatic incident. It discusses pneumopericardium’s causes, symptoms, and complications, including tamponade symptoms and imaging modalities, to confirm the diagnosis and assess complications. Present various treatment options emphasize the importance of ongoing monitoring and damage control principles. **Methods:** Retrieved pneumopericardium cases with complete clinical databases (Pubmed, Medline) and summarised the clinical symptoms and prognosis of different treatment schemes. **Results:** A 33-year-old male patient, after a suicide attempt, fell from a height estimated at 15 m. The patient was intubated at the scene and mechanically ventilated. Initial assessment revealed signs of tension pneumothorax, which was decompressed and stabilized. Subsequent deterioration led to the discovery of pneumopericardium through diagnostic imaging. **Conclusions:** The case highlights the need for a comprehensive and multidisciplinary approach to address the complex nature of injuries associated with pneumopericardium. It examines the principles of damage control, which focus primarily on the patient’s general condition, leaving some injuries for later treatment.

## 1. Introduction

Pneumopericardium is the presence of air or gas around the heart in a pericardial cavity [1,2,3]. It is a scarce medical condition, in most cases self-limiting, without severe symptoms, and it does not demand treatment. However, in one-third of cases, pneumopericardium can lead to increased pressure in the pericardial sac [2,4,5], an impairment of cardiac output, and symptoms of cardiac tamponade, which is an acute life-threatening condition. The causes can be divided into traumatic and non-traumatic (iatrogenic, asthma crises, fistula, but also rare miscellaneous cases, such as the case of pneumopericardium in paraquat intoxication [6]). According to Cummings et al. [2], in traumatized patients, pneumopericardium is more common in blunt trauma than penetrating trauma (93.25% vs. 6.75% of patients with pneumopericardium).

The presence of air in the pericardium has various pathophysiological causes: air leakage from the pleural cavity into the pericardial cavity through a small tear in the pleura and pericardium in pneumothorax and direct leakage of air from the ruptured trachea or bronchus or the Macklin effect when air penetrates along the pulmonary veins from the rupture of the respiratory tract, which dissects peri bronchial–vascular sheaths passing to the pericardium [1,7]. The use of positive-pressure mechanical ventilation can push air through perivascular sheaths to the pericardial cavity, and the pericardium acts as a valve, preventing the leak of air and leading to circulatory compromise [8]. Sometimes, the symptoms can occur after the initiation of positive-pressure mechanical ventilation [9,10]. In the literature, there are also reports that air can leak into the pericardial cavity through the venous sub-adventitia in case of an open injury of the veins of the throat or during venous cannulation and catheterization [11].

The rarity of pneumopericardium is associated with the small size of the critical sites for pneumopericardium formation and the relatively protected position of the heart in the thorax. Nevertheless, when it does occur, it often coexists with damage to large vessels, bilateral pneumothorax, or pneumomediastinum, which can result in sudden death at the scene [5,12]. Although being a relevant cause of shock in trauma, pneumopericardium is rarely regarded during initial trauma evaluation. Initially, every pneumopericardium is a non-tension pneumopericardium, and the clinical picture of pneumopericardium in a trauma patient can be misleading due to the overlap of additional injuries, and any single one can lead to shock. Classic symptoms of pneumopericardium include paradoxical pulse, tachycardia, increased central venous pressure, Hamman sign, or bruit de moulin—a characteristic crunching murmur synchronized with the heartbeat [13].

## 2. The Aim of the Case Description

We analyzed the clinical symptoms and treatment of a 33-year-old male patient with pneumopericardium admitted to the hospital. We retrieved pneumopericardium cases with complete clinical data in PUBMED, MEDLINE, and other databases for analysis, and we summarized the clinical symptoms and prognosis of different treatment schemes.

## 3. The Case Description

On 11 July 2023, at 7:40 p.m., the Emergency Department received information about a 33-year-old male patient who fell from a height estimated at 15 m. The time to reach the Emergency Department was about 8 min. He was intubated without sedation at the scene due to unconsciousness, mechanically ventilated with SIMV, FiO_2_ 60%, PEEP 5 cm H_2_O without sedation, and GCS (Glasgow Coma Scale): V—non-testable, E—1, and M—3 [14]. On admission, he presented decreased breath sounds and subcutaneous emphysema on the right side of the thorax and neck, a peripheral saturation of 80%, a heart rate of 60 bpm, a blood pressure of 97/70 mmHg, a breathing rate of 30/min, and an abrasion of the epidermis on the right side of the forehead; a deep wound was seen on the right arm with deformation on the distal arm. In the second intercostal space, he had a needle to decompress the pneumothorax without the sound of air flowing through the needle. The Revised Trauma Score was 3.2744 points. The eFAST protocol (Extended Focused Assessment with Sonography for Trauma) revealed a lack of sliding in the second to fourth intercostal space in the mid-clavicular line, and the view on the Morison pouch and pericardial view was impossible to assess—the A and E artifacts were visible [15]. The critical parameters from the blood gas analyzer are as follows (7:54 p.m.): pH 7.102, pCO_2_ 49.7 mmHg, pO_2_ 138 mmHg, HCO_3_ 15.5 mmol/L, BE −14.2, anion gap 20.1 mmol/L, hemoglobin 13.4 g/dL, glucose 200 mg/dL, and lactate 7.3 mmol/L.

Due to the suggestive symptoms, the tension pneumothorax was immediately decompressed, and a thoracic drain 32F was placed into the suitable pleural space, obtaining a dynamic outflow of air without signs of bleeding from the pleural cavity. The needle from the anterior chest wall was removed. After the procedure, the patient was hemodynamically stable; his blood pressure was 125/90 mmHg, his heart rate was 105 bpm, and his peripheral saturation was 98%. He began responding; GCS: V—non-testable, E—3, and M—3, and sedation with midazolam was applied.

After 15 min, a gradual decrease in blood pressure was observed. A bedside ultrasound was performed according to the RUSH protocol (Rapid Ultrasound for Shock and Hypotension), but we could not assess the heart from any of the possible ultrasound windows. The second examination of critical parameters revealed the following (8:56 p.m.): pH 7.067, pCO_2_ 53 mmHg, pO_2_ 371 mmHg, hemoglobin 10.5 g/dL, glucose 118 mg/dL, and lactate 7.6 mmol/L. Due to a significant decrease in hemoglobin (2.9 g/dL in an hour) and increased lactate level, we began a transfusion of the red blood cells.

**Figure 1:**
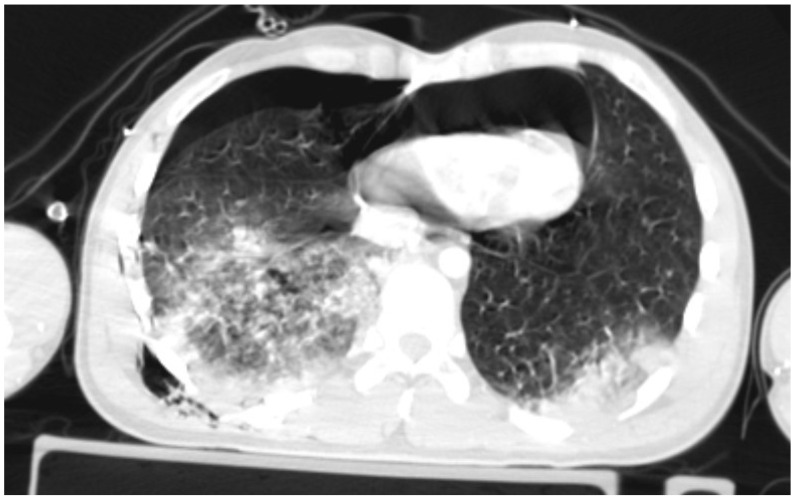
Pneumothorax and pneumopericardium with features of cardiac tamponade.

A CT (Computed Tomography) scan showed right-sided pneumothorax with a chest drain (the maximum width of pneumothorax 20 mm) and a pneumopericardium (max. 30 mm) with the symptoms of compression of the anterior wall of the heart. There were features of cardiac tamponade (Figure 1); there was a right lower lobe contusion and rib fractures from V to XII on the right side (some of the ribs fractured in multiple places with displacement); a right hemothorax up to 20 mm; a para cerebral hematoma in the left temporal region up to 3 mm; numerous sites of retroperitoneal bleeding along the iliac vessels (on the right side up to 30 mm in diameter), hematoma around the bladder and right iliopsoas muscle; a small hypodense area alongside to the right kidney up to 10 mm with a hypodense area adjacent to the liver up to 16 mm; a contusion of the soft tissues of the right buttock with hematoma (max. 30 mm); multiple fractures of the pelvis; a collapsed inferior vena cava; and an open fracture of the right elbow. The Injury Severity Score had a value of 50 points.

The patient’s condition after a CT scanning was critical, with an arterial pressure of 40/0 mmHg, saturation undeterminable, and a heart rate of 40 bpm. Decompression of the tension pneumopericardium was performed with the use of a Veres needle—an ultrasound control during the puncture was ineffective due to the image of air covering the echo of the needle. It was decided to puncture from the fourth intercostal space at the anterior axillary line with a syringe with saline attached to measure the amount of decompressed air—about 50 mL of air was obtained.

**Figure 2:**
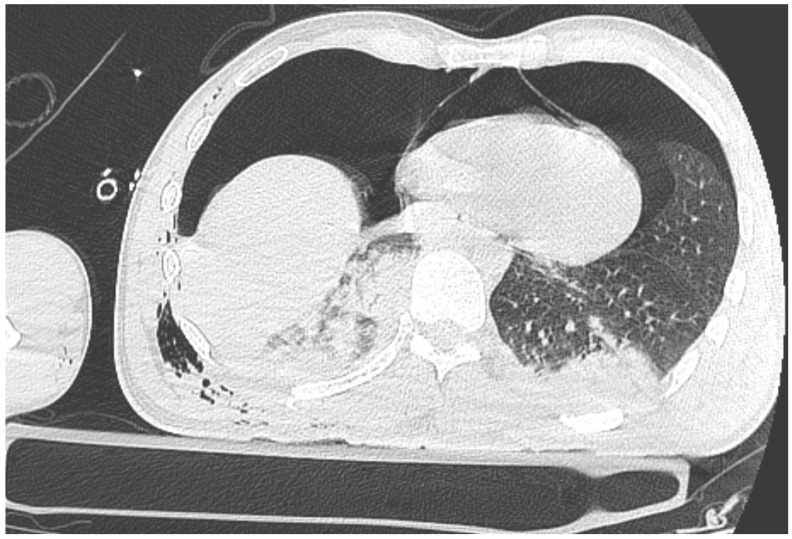
CT scan of pneumothorax and pneumocardium after a pneumopericardial decompression—scan at the level of the diaphragm.

**Figure 3:**
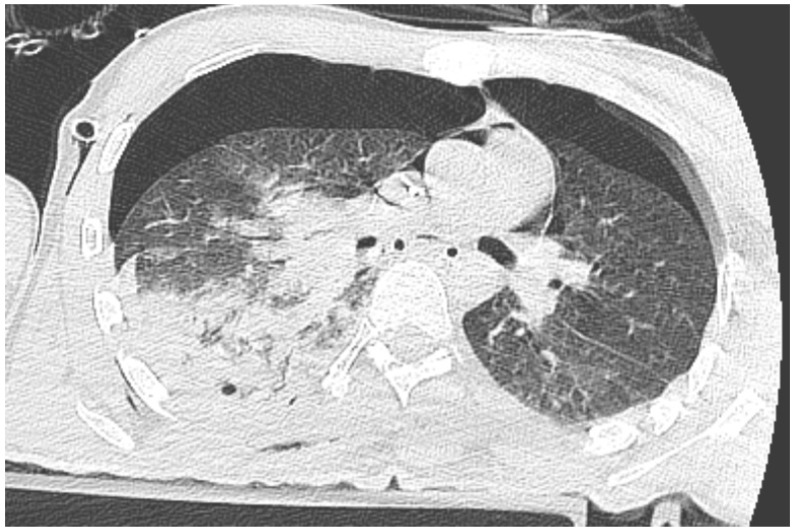
CT scan of pneumothorax and pneumocardium after pericardial decompression of the pneumopericardium.

Immediately after decompression, the blood pressure was 134/85 mmHg, the heart rate was 105 bpm, and the saturation was 92%. The critical parameters from the blood gas analyzer just after the decompression of pneumothorax are as follows (11:15 p.m.): pH 7.016, pCO_2_ 45.2 mmHg, pO_2_ 107 mmHg, HCO_3_ 11.6 mmol/L, BE −19.4, anion gap 19.4 mmol/L, and hemoglobin 15.0 g/dL (after transfusion of three units of red blood cells). Laboratory blood tests showed a level of troponin 15,158.657 pg/mL. In the next 30 min, the blood pressure was 85/50 mmHg. Catecholamine infusion was initiated, fluid therapy was continued, and point-of-care ultrasound diagnostics were repeated, Still, we could not obtain the heart from any ultrasound window. A new CT scan was performed with no contrast, and there was bilateral pneumothorax small pneumopericardium in the pericardial cavity (Figure 2 and Figure 3). Drainage of the left pleural cavity was performed. The right drain was oscillating, but it was no longer functional and was trapped in the lung fissure between the expanding lobes of the right lung, which was seen on CT scans. The right pleural drain was replaced, which resulted in an improvement in the patient’s condition. However, he still required the use of catecholamine.

Due to the fluid around the liver without any signs of leaking of contrast from the liver in CT, it was decided to perform a diagnostic peritoneal lavage to prove that there was no active bleeding in the abdomen and that the presence of the fluid was caused by the contusion of the liver—without signs of active bleeding. The patient was admitted to the ICU (Intensive Care Unit) for further treatment. In the ICU, further growth of troponin was observed (30,060.298 pg/mL). In the following days, he was consulted by the cardiologist. At that time, the pericardium was without pathological fluid or air, and the ejection fraction was 35–40%. Due to the symptoms of kidney failure, he required dialysis. A right pleural drain was removed on the 20th day and the drain from the left pleural cavity on the 29th day. On the 30th day, the patient was conscious. The last time he required dialysis was on the 48th day, and he was discharged from the hospital after the next 35 days due to orthopedic treatment and rehabilitation.

## 4. Discussion

Pneumopericardium in adult patients, in most cases, is caused by blunt trauma, in which motor vehicle accidents, depending on the literature, account for 72.2% [16] to 94% of cases [17], whereas the second most common cause of pneumopericardium is falling from height. The prevalence of pneumopericardium increases with height [5], and in the cases of falls from a height of 10 m or more, there is a 90% risk that the patient has a pneumothorax or hemothorax. The ruptures of the pericardium are extremely rare; 50–64% of them are in the left pneumocardial region, and 9–17% are in the right pneumocardial region [18]. Kamiyoshihara et al. [19] described a patient crushed by a car with bilateral pneumothorax and pneumopericardium, from whom the authors deduced the pericardial rupture. As suggested by the more significant pneumothorax on the left side, they assumed that the pericardial tear occurred on the same side. During the operation, it was revealed that the tear exists on the right side. The authors of this case report stated that the expanse of the pneumothorax cannot necessarily reliably identify the side of the ruptured pericardium. To determine the side of a pericardial tear, they suggest performing chest drainage—if the drain is on the ruptured side, then the pneumomediastinum or the pneumopericardium will decrease; otherwise, they will remain unchanged. Our patient, despite the right-sided pleural drainage, had features of the tension pneumopericardium, which allowed us to rule out the pericardial tear as a cause of the pneumopericardium.

### 4.1. The Diagnosis

**Figure 4:**
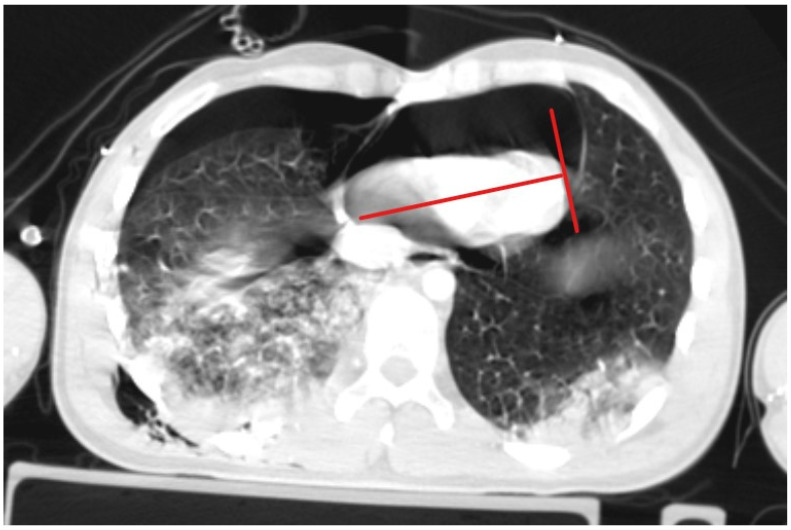
Ballooning is a sign of tension pneumopericardium.

A patient with blunt trauma may have several severe injuries; each of them, if untreated, can be fatal. A post-mortem examination of patients after falls from heights did not reveal the presence of isolated pneumopericardium; it was always accompanied by other severe injuries [5]. The clinical picture of increasing pneumopericardium may be confused by concurrent conditions: cardiac contusion, pneumothorax, hemorrhage, or spinal shock [20]. Initially, our patient presented symptoms that could suggest both tension pneumothorax and tension pneumopericardium. Bedside ultrasonography revealed two significant symptoms: the absence of pleural sliding on the right side of the chest and the lack of heart image in the available ultrasound imaging windows. Cheryl L. Reid et al. [21] in 1983 described the characteristic signs of pneumomediastinum and pneumopericardium as an “air gap sign”. This symptom involves the cyclic appearance of air echoes, thus covering the image of the heart during systole and early phase of diastole of ventricles. It is caused by a physical shift and a reduction in the volume of air due to changes in the volume of the ventricles during the heartbeat [21,22], but this symptom does not occur in cases of tension pneumopericardium because elevated pressure in the pericardium keeps the heart under the layer of air. Due to the more frequent occurrence of tension pneumothorax compared to tension pneumopericardium, drainage of the right pleural cavity was performed, achieving the initial improvement in the patient’s condition, which allowed for performing CT scanning in the polytrauma protocol, where symptoms of tension pneumopericardium were seen. Heimer et al. [5] stated “ballooning” as a typical sign of tension pneumopericardium in CT scanning, which involves the protrusion of the pericardium beyond the line drawn of the apex of the heart and perpendicular to its axis, as shown in Figure 4. If the pericardium does not pass beyond this line, the pneumopericardium is considered normotensive.

The occurrence of hemodynamic disorders in the patient with pneumopericardium obliges to decompress the pericardium in an invasive way immediately due to the fast-deteriorating condition of the patient, and it was decided to perform pericardiocentesis. Stegmaier et al. believe that pericardiocentesis is a procedure that can be used in an emergency but is not suitable for definite treatment [23]. Moore et al. suggest that landmark-guided pericardiocentesis is a viable option for treatment in resource-limited settings [24]. Gołota et al. recommend that in the case of cardiac tamponade, a pericardial decompression can be achieved by needle, incision, or pericardial drainage. They also recommend access from the area of the costal angle below the xiphoid process, and this method should be supplemented with antibiotic therapy [25]. Cummings et al., in adult patients with pneumopericardium with ventilator therapy, suggest an initial pericardiocentesis followed by pericardial drainage under vision control [2]. Ladurner et al. state that each patient after pericardiocentesis should be transported to the operating room for pericardial drainage [20].

### 4.2. Treatment Options

**Figure 5:**
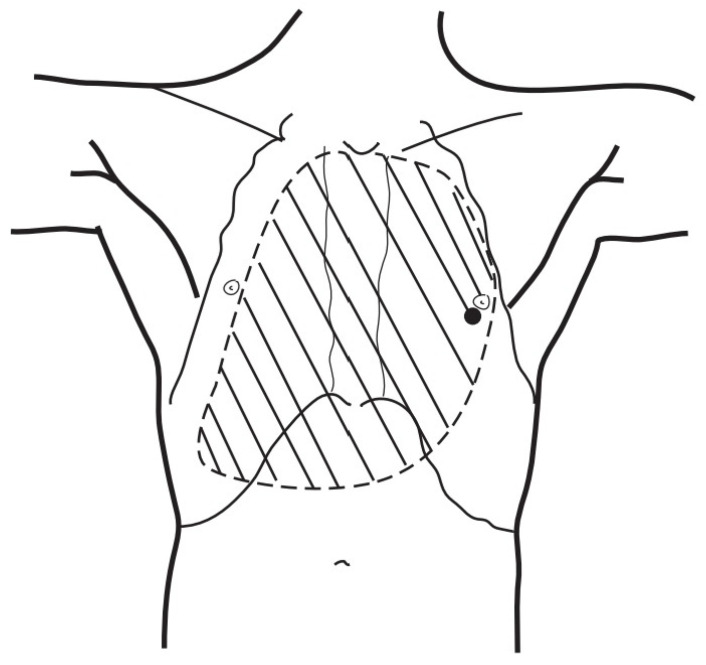
The area of the lack of sliding in lung ultrasonography and the point of needle decompression of tension pneumopericardium.

In our patient, the pericardiocentesis was performed in the precordial region, at the border of the image of the lung and the static image of air (the lack of pleural sliding, barcode image in M-mode), which corresponded to the IV intercostal space, directing the needle to the right shoulder according to the fourth intercostal space at the anterior axillary line (Figure 5).

**Figure 6:**
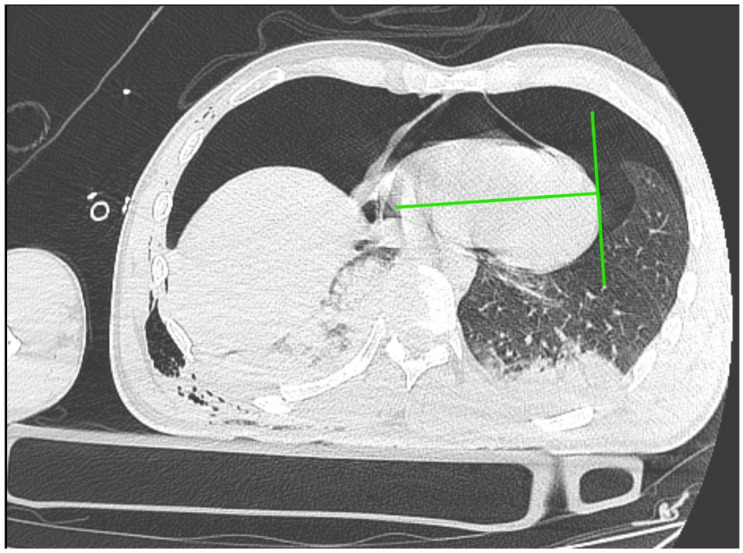
Pneumopericardium without signs of tension pneumopericardium.

Approximately 50 mL of air was obtained, which resulted in a significant reduction in symptoms despite the persistence of pneumopericardium in the CT scan but without the signs of elevated pressure in the pericardial sac (Figure 6).

Usually, the symptoms of tension pneumopericardium occur once the pressure in the pleural cavity exceeds 145 mm H_2_O [22], which is equivalent to 60 mL of air. In our case, the pericardiocentesis caused the penetration of air in the left pleural cavity and the rise of left pneumothorax, the drainage of which effectively drained air from the pericardial cavity. This confirms the theory of Kamiyoshihara et al. about the possibility of air drainage through a drain placed in the pleural cavity on the side of the pericardial tear—in our case, this was an iatrogenic tear transforming pneumopericardium in the left pneumothorax [19]. Due to the patient’s critical condition and numerous injuries, including bleeding, pelvic fracture, and multi-causal shock, it was decided to implement damage control procedures, and the decision of the final treatment was left for the later period of therapy. Bronchoscopy performed in the following days did not reveal bronchial rupture, and echocardiography showed no accumulation of air or fluid in the pericardial cavity, which allowed the resignation of performing pericardial drainage. Esophagoduodenoscopy was discontinued due to the absence of pneumomediastinum.

## 5. Conclusions

Pneumopericadium is a rare condition that can turn into a life-threatening disorder. In the case of trauma, it is tough to manage in the first hours of care due to the overlap of the injuries from different systems. In our patient, we initially observed shock from the proper tension pneumothorax, followed by the tension pneumopericardium, cardiac contusion with cardiogenic shock, and the loss of blood from the fractures of the pelvis, leading to hypovolemic shock. It is essential to keep in mind the principles of damage control, which focus primarily on the patient’s general condition, leaving some injuries for later treatment if immediate treatment is a burden that leads to the patient’s death. Decompression of pneumopericardium in the emergency department with a needle may not be sufficient, and surgical management should be considered, bearing in mind the cost–risk assessment for the patient. Many researchers have noticed that the development of pneumopericardium and further cardiac tamponade can occur with a delay of up to 10 h [23], often after starting mechanical ventilation, increasing positive pressure in airways [2,4,23,26]. In these cases, it is essential to be aware that the absence of an initial pneumopericardium does not eliminate the risk of subsequent occurrence, even if mechanical respiratory support does not occur.

## Data Availability

The original contributions presented in the study are included in the article; further inquiries can be directed to the corresponding author.
